# Analysis of combination therapy of immune checkpoint inhibitors in osteosarcoma

**DOI:** 10.3389/fchem.2022.847621

**Published:** 2022-09-06

**Authors:** Lijun Peng, Huapan Fang, Xiao Yang, Xi Zeng

**Affiliations:** ^1^ Hunan Province Key Laboratory of Tumor Cellular and Molecular Pathology, Cancer Research Institute, Hengyang Medical School, University of South China, Hengyang, China; ^2^ The First Affiliated Hospital, Department of Spine Surgery, Hengyang Medical School, University of South China, Hengyang, China; ^3^ Cancer Epigenetics Laboratory, Department of Clinical Oncology, State Key Laboratory of Oncology in South China, Sir YK Pao Center for Cancer and Li Ka Shing Institute of Health Sciences, The Chinese University of Hong Kong, Hong Kong, Hong Kong, SAR China; ^4^ Institute of Functional Nano and Soft Materials (FUNSOM), Jiangsu Key Laboratory for Carbon Based Functional Materials and Devices, Soochow University, Suzhou, China; ^5^ Hong Kong Centre for Cerebro-Caradiovasular Health Engineering (COCHE), Hong Kong, Hong Kong, SAR China

**Keywords:** immunotherapy, PD-1, PD-L1, CTLA-4, sarcoma, osteosarcoma

## Abstract

One of the most common primary bone malignant tumors is osteosarcoma (OS), possessing a high tendency of local invasion and distant metastasis. Although surgery combined with chemotherapy can extend the patients’ survival time, the prognosis for most patients with metastases or relapses is poor. Immunotherapy has been proved to be a promising treatment alternative for malignant tumors. Although there is a significant amount of animal- and cell-based evidence supporting the relationship between immune checkpoint inhibitors (anti-PD-1, anti-PD-L1, anti-CTLA-4) and cancers, no pan-cancer analysis is available. Simultaneously, immune checkpoint inhibitors have demonstrated satisfactory clinical results in some tumors; however, only a small fraction of patients with certain cancer types have been benefitted. Therefore, based on the TCGA dataset, we first explored the potential roles of immune checkpoints in 33 tumors. Programmed death receptor 1 (PD-1), programmed cell death ligand 1 (PD-L1), and cytotoxic T lymphocyte antigen 4 (CTLA-4) were not consistently expressed in the same direction in all tumors; however, the direction of expression change was the same in most immune cells. Although there is no well-established relationship between the expression of PD-1/PD-L1/CTLA-4 genes and the prognosis of patients with sarcomas, their interaction and extent of immune cell infiltration into sarcomas are probably the key determinants of therapeutic response. Our first pan-cancer study provides a relatively comprehensive understanding of immune checkpoint inhibitors in different sarcomas.

## 1 Introduction

Sarcomas (SARCs) are primarily divided into soft tissue and bone, originating from mesenchymal stem cells (Wunder et al., 2007). In general, SARCs include leiomyosarcoma, lymphosarcoma, synovial sarcoma, and osteosarcoma (OS). OS is one of the most common aggressive bone tumors in children and adolescents (Jafari et al., 2020). It primarily occurs in the metaphysis of long tubular bones such as the proximal humerus, distal femur, and proximal tibia (Norregaard et al., 2021). The hallmark clinical symptoms of OS include local pain and swelling and occasionally joint dysfunction. It accounts for 20% of clinical cases of malignant bone tumors worldwide (Lilienthal and Herold, 2020). OS is considered to be a critical threat to human health globally owing to its high malignancy, strong invasiveness, rapid progress, frequent recurrence, and high mortality rates. Moreover, OS has a high tendency of early metastasis, with the common site being the pulmonary parenchyma (Kager et al., 2003).

The primary treatment for osteosarcoma was surgery (Siegel and Pressey, 2008) and the patients generally underwent amputation in the past. Limb salvage surgery has gradually replaced the traditional amputation with the development of surgical techniques and chemotherapeutic drugs. In 2019, the European and American Osteosarcoma study-1 followed up more than 2000 patients with high-grade osteosarcoma from April 2005 to June 2011 and concluded that for the complete surgical resection of all sites, combined with methotrexate, doxorubicin, and cisplatin as standard chemotherapy, the 3- and 5-years event-free survival rates were 59% and 54%, respectively (Smeland et al., 2019). Currently, approximately 68% of patients with localized OS have a survival rate of 5 years or more (Siegel et al., 2018); however, approximately 20%–30% survive in metastatic or recurrent cases (Jafari et al., 2020; Chen et al., 2021). The occurrence of metastatic tumors is an obvious sign of poor prognosis of OS.

Current chemotherapy still depends on a combination of four different drugs (methotrexate, doxorubicin, cisplatin, and ifosfamide) (Ferrari and Serra, 2015; Bielack et al., 2016). To some extent, the low survival rate of patients with OS is attributed to chemotherapy resistance and side effects. Current therapies often encounter the bottlenecks of chemotherapy resistance and tumor immune escape (Lilienthal and Herold, 2020). Therefore, it is urgently needed to develop a novel strategy for OS treatment.

Immunotherapy as a new therapeutic method has modified the field of oncology by human immune response activation (Robbins and Morelli, 2014). Immune checkpoints as immunosuppressive molecules can regulate the intensity and breadth of the immune response (Pardoll, 2012). During tumorigenesis and development, immune checkpoints such as programmed death receptor 1 (PD-1), programmed cell death 1 ligand 1 (PD-L1), and cytotoxic T lymphocyte antigen 4 (CTAL-4) have become one of the primary causes of immune tolerance (Pardoll, 2012; Fang et al., 2021; Chen et al., 2022). CTLA-4 and PD-1 are the main inhibitory receptors expressed on T cells, and they act as inducing signals to prevent T cell overactivation (Leone and Powell, 2020). PD-1, PD-L1, and CTLA-4 are the primary immune checkpoint molecules (Pardoll, 2012). Blocking CTLA-4 and PD-1 can improve the prognosis of some cancers, including melanoma, non-small cell lung cancer, and renal cell carcinoma (Brahmer et al., 2012; Topalian et al., 2012). Therefore, novel treatment strategies to block these inhibitory receptors appear to show great potential for malignant tumors.

Owing to the complexity and features of tumorigenesis, the levels of pan-cancer relevant genes must be analyzed, and the correlation between clinical prognosis and potential molecular mechanism must be evaluated. However, according to the clinical database, there is no pan-cancer analysis available for PD-1, PD-L1, and CTLA-4. Therefore, based on various databases, this study analyzed the expression of PD-1, PD-L1, and CTLA-4 in patients with pan-cancer and sarcoma. The correlation between clinicopathological features and prognosis of patients with osteosarcoma and its potential regulatory mechanism is further analyzed.

## 2 Materials and methods

### 2.1 Gene structure and expression analysis

We used the Gene database (https://www.ncbi.nlm.nih.gov/gene/) and UniProt database (https://www.uniprot.org/), enter PD-1, PD-L1, CTLA-4, respectively, to obtain genes information and structure. We used the online mapping tool (http://www.wormweb.org/exonintron) to input the 5′UTR, 3′UTR, exon-introns of the genes, and obtained simple structure diagrams of the full-length gene transcript. We used IBS (Bio Sequence Illustrator) tool (http://ibs.biocuckoo.org/) to map the protein domain according to the information from the Uniport database.

Then, we inputted PD-1, PD-L1, and CTLA-4 into the “Gene DE” module of the Tumor Immunity Assessment Resources, Version 2 (TIMER2) network (http://timer.cistrome.org/) to obtain PD-1, PD-L1, and CTLA-4 expression in pan-cancer of the TCGA project. We used the UALCAN database (http://ualcan.path.uab.edu/analysis.html) to obtain the box diagrams of the differences in the expression of PD-1, PD-L1, and CTLA-4 in sarcoma and corresponding normal tissues.

### 2.2 Genetic alteration analysis

After logging on to the cBioPortal website (https://www.cbioportal.org/), we selected the “TCGA Pan-Cancer Atlas Studies” in the “Quick selection” section and entered PD-1, PD-L1, and CTLA-4 to search for genetic changes. The results of the alteration frequency, mutation type, and copy number alteration (CNA) across all TCGA tumors were observed in the “OncoPrint” module. The mutated site information can be displayed in the schematic diagram of the protein structure or the three-dimensional (3D) structure via the “Mutations” module. We also used the “Comparison/survival” module to obtain the data on the overall and disease-free survival differences for the SARC samples of the TCGA database with or without PD-1, PD-L1, and CTLA-4 genetic alteration. Kaplan-Meier plots with log-rank *p*-value were generated as well.

### 2.3 Survival prognosis analysis

We used the “TCGA” module of the UALCAN database (http://ualcan.path.uab.edu/analysis.html) to obtain survival probabilities of the expression high and low/medium expressions of PD-1, PD-L1, and CTLA-4 in sarcoma.

### 2.4 Immune infiltration analysis

The genes expression in immune cells data of sarcoma level 3 in HTSeq-FPKM were downloaded from the TCGA database for analysis. The R software (version 4.1.1, https://www.r-project.org/) was used in this analysis. We applied the “GSVA” (https:// www.bioconductor.org/packages/3.4/bioc/htmL/GSVA.html) R package to apply for immune infiltration estimations in sarcoma and includes 24 tumor-infiltrating lymphocytes (activated dendritic cell [aDC], B-cell, CD8 T-cell, cytotoxic cell, DC, eosinophils, inactive DC [iDC], macrophages, mast cell, neutrophils, NK CD56 bright cell, NK CD56 dim cell, NK cell, pDC, T helper cell, T-cell, T central memory [Tcm], T effector memory [Tem], T follicular helper [Tfh], Tgd, Th1 cell, Th17 cell, Th2 cell, and Treg) (Bindea et al., 2013).

### 2.5 Co-expressed genes of PD-1/PD-L1/CTLA-4 analysis

The “Gene _ Corr” module of the TIMER2.0 method was used to analyze the expression correlation of PD-1, PD-L1, CTLA-4 in pan-cancer and demonstrated as a heatmap, which contains the partial correlation (cor) and *p*-value in the purity-adjusted Spearman’s rank correlation test. We also used the “Gene _ Corr” module of the TIMER2.0 approach to analyze the expression Pearson’s correlation between PD-1, PD-L1, CTLA-4 in sarcoma from TCGA. The *p* value and the correlation coefficient (r) were indicated.

To analyze the co-expression of these three genes, we also downloaded TCGA (https://portal.gdc.cancer.gov/) SARC (sarcoma) project level 3 HTSeq-FPKM format RNAseq data. The significant score was NS, *p* ≥ 0.05%, *, *p* < 0.05, **, *p* < 0.01, ***, and *p* < 0.001. Software: R (version 4.1.1), R package: mainly “ggplot2” (https://cran.rproject.org/web/packages/ggplot2/index.html). Then, we used the interactive Venn diagram viewer (https://bioinfogp.cnb.csic.es/tools/venny/index.html) to perform the intersection and co-expression of PD-1, PD-L1, and CTLA-4 genes.

### 2.6 Related gene enrichment analysis

We first searched the STRING website (https://string-db.org/) using the query of a single protein name (“PD-1, PD-L1, CTLA-4”) and organism (“*Homo sapiens*”). Subsequently, we set the following main parameters: minimum required interaction score (“Medium confidence [0.40]”), the meaning of network edges (“evidence”), max number of interactors to show (“no more than 10 interactors” in the first shell) and active interaction sources (“experiments”). Finally, the available experimentally determined PD-1, PD-L1, CTLA-4-binding proteins were obtained.

Based on the PD-1/PD-L1/CTLA-4-binding and interacted co-expressed genes, to further comprehend the potential role of PD-1, PD-L1, CTLA-4 in sarcoma, Gene Ontology (GO) and Kyoto encyclopedia of genes and genomes (KEGG) analyses were performed. The enriched pathways were finally visualized with the “cluster Profiler” (https://www.bioconductor.org /packages/release/bioc/htmL/clusterProfiler.html) and “ggplot2” (https://cran.r-project.org/web/packages/ggplot2/index. html) R packages. The GO data for biological process (BP), cellular component (CC), and molecular function (MF) were visualized.

## 3 Results

### 3.1 Gene structure analysis data

**FIGURE 1:**
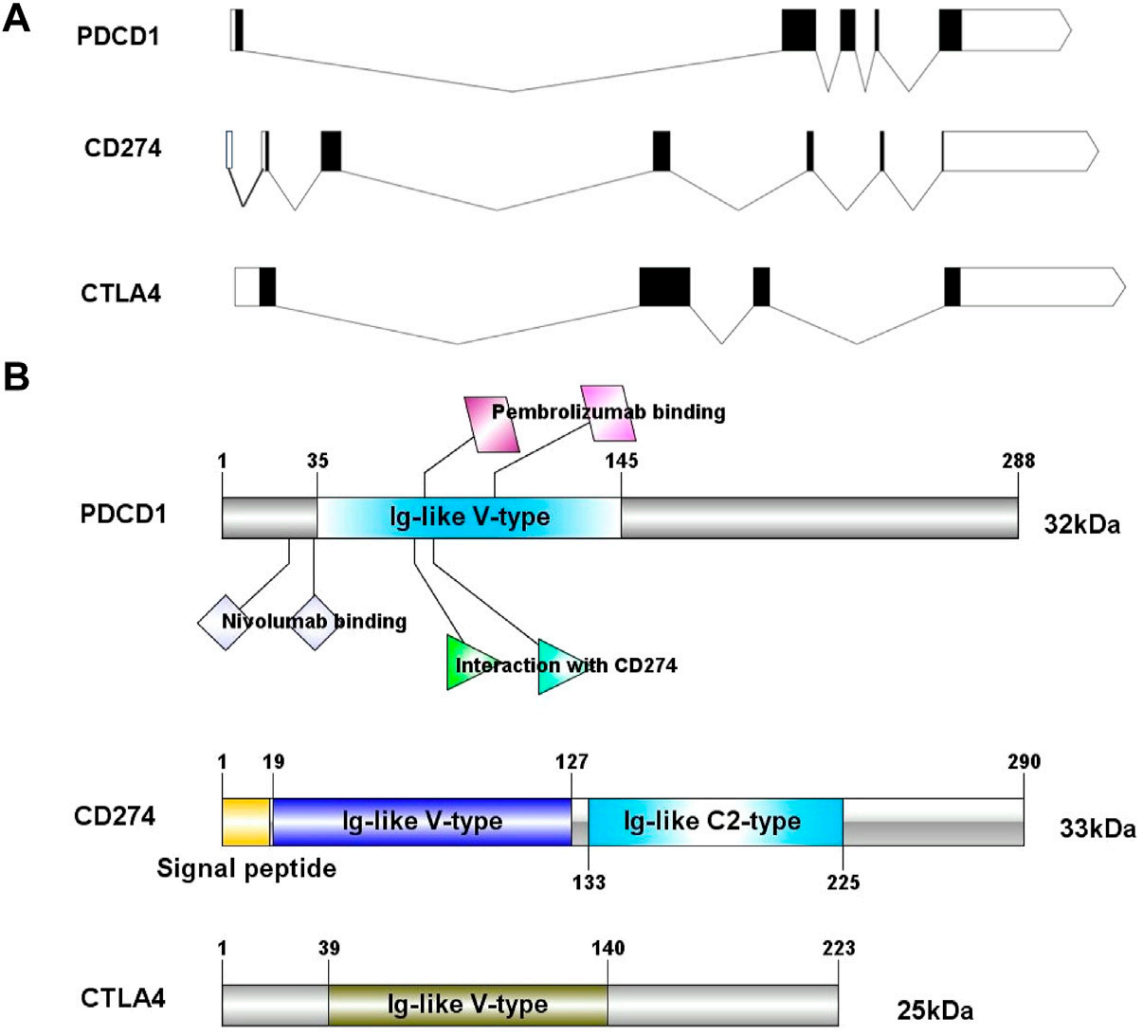
The exon-intron structure of PD-1/PD-L1/CTLA-4 genes **(A)** with domain structure of the corresponding protein **(B)**. **(A)** Exons are designated by black rectangles, 5′UTR and 3′UTR -by white rectangles, introns-by horizontal lines. **(B)** Length for PD-1/PD-L1/CTLA-4 protein (aa) and colored blocks represent protein domains. PD-L1 contains Ig-like V-type domain and Ig-like C2- type domain, PD-1 and CTLA-4 only contain Ig-like V-type domain.

In this study, we obtained the basic gene and protein information of PD-1, PD-L1, and CTLA-4 through the gene bank and UniProt databases (Table 1). We drew a map of the full-length transcript and domains of these three genes (Figure 1). We found that they all contain Ig-like V-type domains, and both PD-1 and CTLA-4 are located on the 2p chromosome, which implies that their functions and mechanisms have a certain degree of similarity.

**TABLE 1:** Basic gene information and protein information of PD-1, PD-L1, CTLA-4.

Name (Official full name and symbol)	Aliases	Exon count	Protein name	Protein	Chromosomal location
				Domain	
PD-1 (Programmed cell death 1)	CD279; SLEB2; hPD-1; hPD-l; hSLE1	6	Programmed cell death protein 1	Ig-like V-type	2q37.3
PD-L1 (CD274)	B7-H; B7H1; hPD-L1; PD-1L1; PD-1LG1	7	Programmed cell death 1 ligand 1	Ig-like V-type	9p24.1
				Ig-like C2-type	
CTLA-4 (Cytotoxic T-lymphocyte associated protein 4)	CD; GSE; GRD4; ALPS5; CD152; IDDM12; CELIAC3	4	Cytotoxic T-lymphocyte protein 4	Ig-like V-type	2q33.2

### 3.2 Genetic alteration analysis data

**FIGURE 2:**
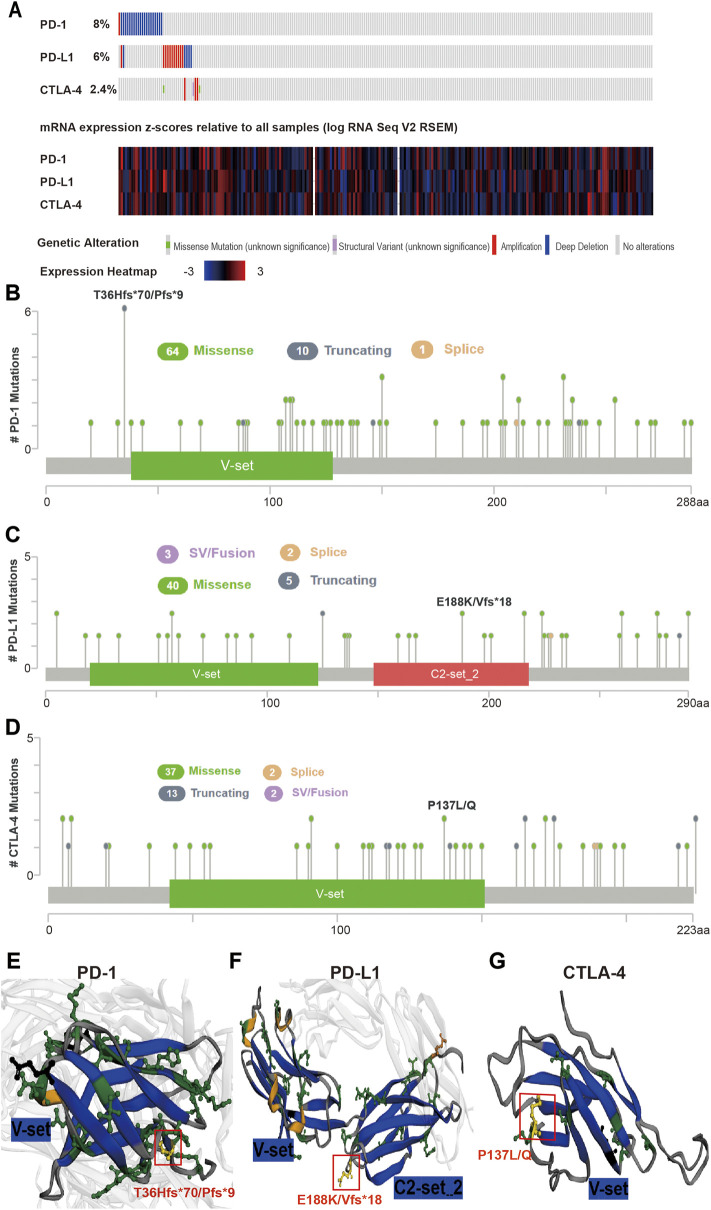
Mutation features of PD-1/PD-L1/CTLA-4 in SARC specimens. **(A)** Genetic alterations and mRNA expression of PD-1/PD-L1/CTLA-4 from all 255 of the human SARC specimens (TCGA, Pancancer Altas) in the TCGA dataset analysed using cBioPortal. A total of 8%, 6%, 2.4% sequenced patients with SARC exhibited genetic alterations of PD-1, PD-L1, CTLA-4. **(B,C,D)** Distribution of **(B)** PD-1, **(C)** PD-L1, and **(D)** CTLA-4 mutation sites in sarcoma. The locations of mutations are shown on the *X*-axis (lollipop) and the frequency of mutations on the *Y*-axis. Different colors represent different types of mutations, and missense mutations are represented by green circles, which occupy the main types of mutations. **(E,F,G)** 3-D protein structure analysis shows the domains of PD-1 **(E)**, PD-L1 **(F),** CTLA-4 **(G)** and the locations of highest frequency mutation sites in PD-1 (T36Hfs*70/Pfs*9), PD-L1 (E188K/Vfs*18), CTLA-4 (P137L/Q) (yellow). Plots were generated using cBioPortal tools (http://www.cBioPortal.org) and curated manually.

**FIGURE 3:**
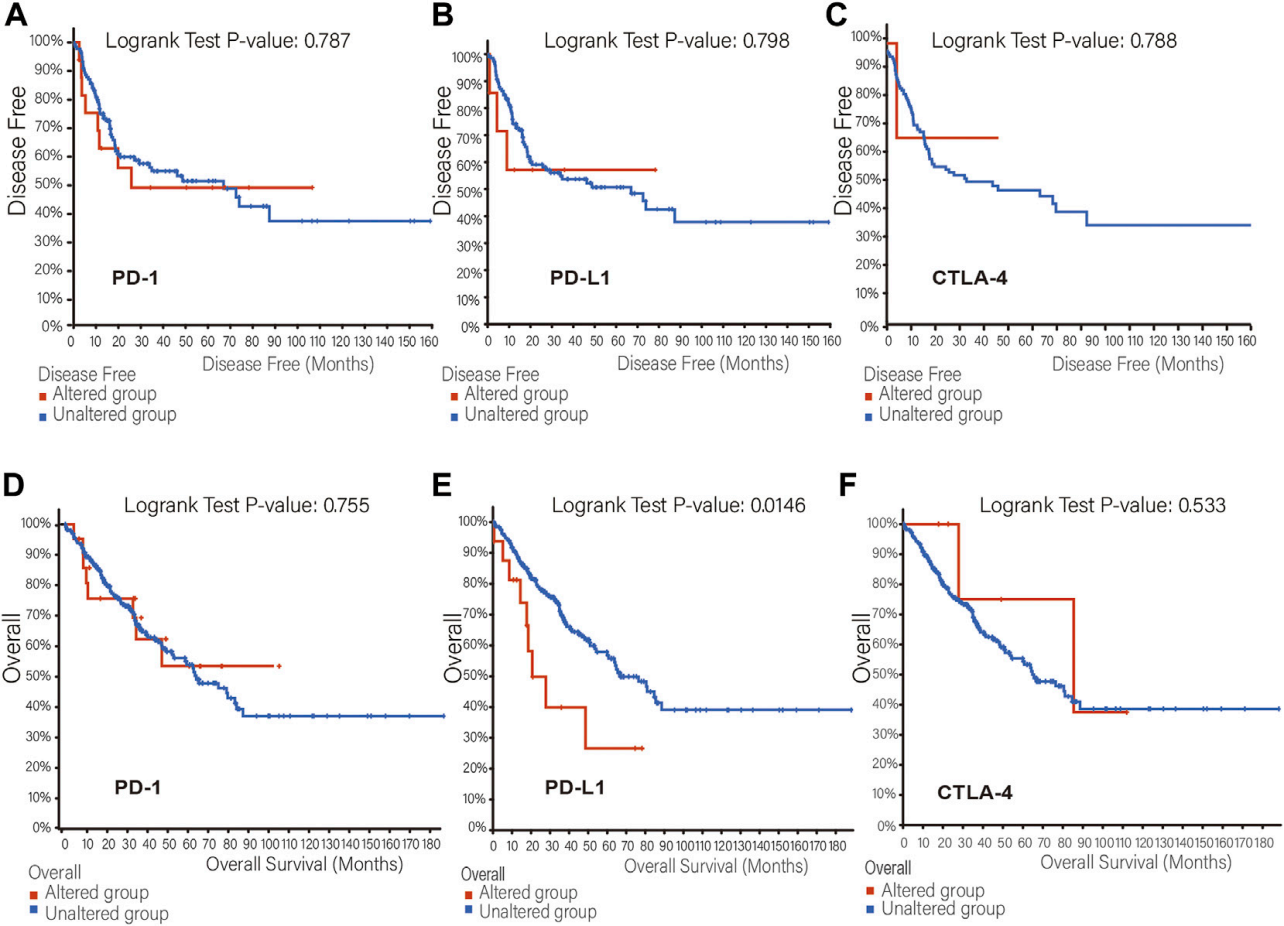
The relationship between mutation frequency and survival. **(A,B,C)** Disease-free survival Kaplan-Meier was performed in cases with or without PD-1 **(A)**, PD-L1 **(B)**, CTLA-4 **(C)** alterations. **(D,E,F)** Overall survival Kaplan-Meier was performed in cases with or without PD-1 **(D)**, PD-L1 **(E)**, CTLA-4 **(F)** alterations.

The mutation frequency of PD-1/PD-L1/CTLA-4 mRNA in 255 human SARC patients from the TCGA database detected by the cBioPortal tool are shown in Figure 2A, with the mutation frequencies of 8%, 6% and 2.4%, respectively. The types, representative cases, and sites of the PD-1/PD-L1/CTLA-4 genetic alteration are further presented in Figures 2B–D. We found that missense mutation of PD-1/PD-L1/CTLA-4 was the main types of genetic alteration. We can observe the mutation sites with the highest alteration frequency (T36Hfs*70/Pfs*9, E188K/Vfs*18, P137L/Q) in the 3D structure of the PD-1/PD-L1/CTLA-4 Figures 2E–G). Additionally, we also explored the potential relationship between PD-1/PD-L1/CTL-A gene changes and the clinical survival prognosis of cases with SARC. Only the PD-L1 gene-altered and unaltered groups had statistical significance in the overall survival of SARC (Figures 3D–F). However, the data of Figures 3A–C indicate that SARC cases with altered or unaltered PD-1/PD-L1/CTLA-4 demonstrated no significant difference in disease-free survival probability (*p* > 0.05).

### 3.3 Gene expression and survival analysis data

**FIGURE 4:**
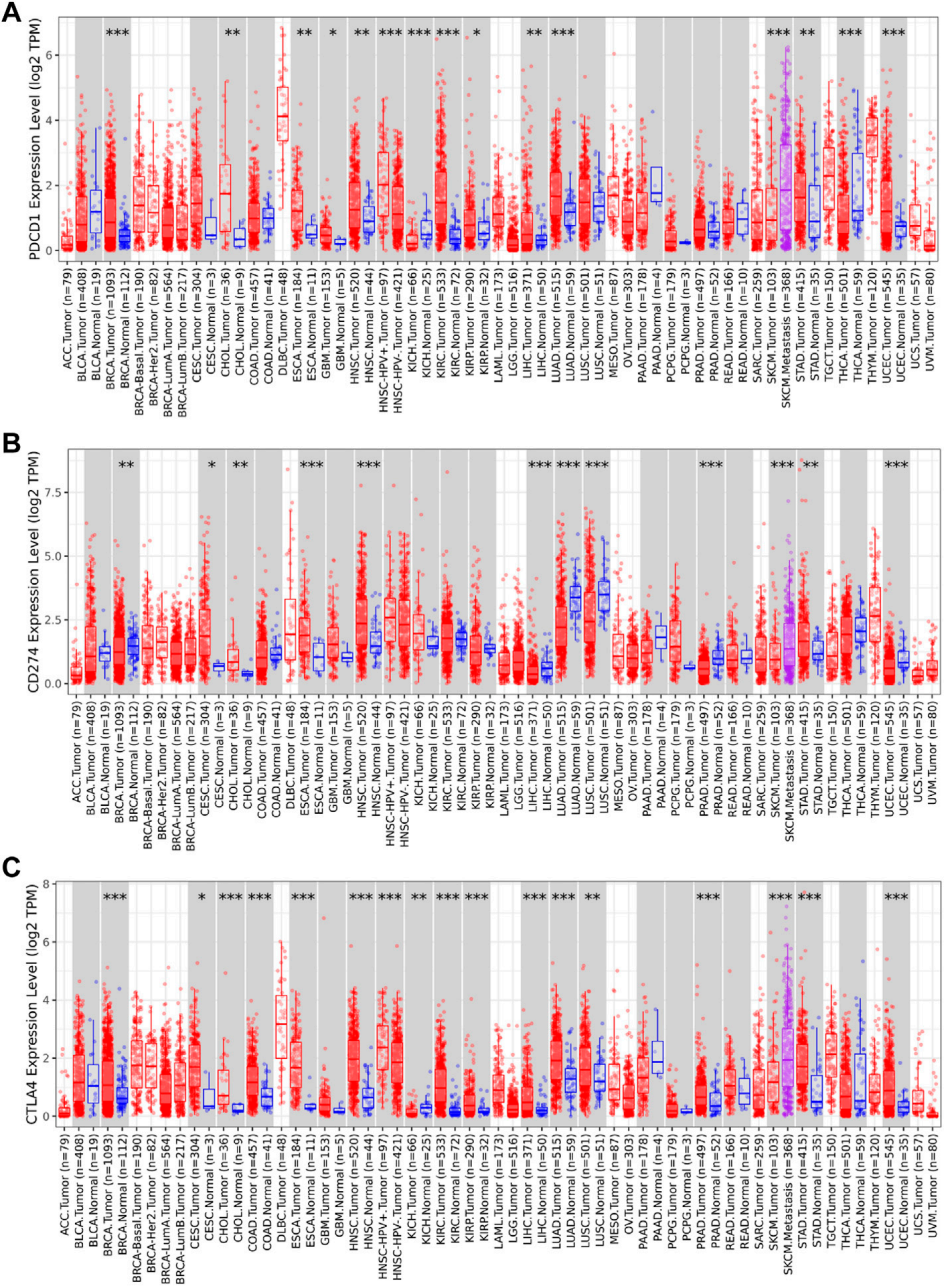
Expression level of PD-1, PD-L1, CTLA-4 genes in different tumors and pathological stages. The expression levels of PD-1 **(A)**, PD-L1 **(B)** and CTLA-4 **(C)** genes in pan-cancer or specific cancer subtypes and paired non-tumor samples from TCGA database was analyzed through TIMER2**. (A,B,C)** PD-1, PD-L1, CTLA-4 showed up-regulation in CHOL (Cholangiocarcinoma), ESCA (Esophageal carcinoma), HNSC (Head and Neck squamous cell carcinoma), and SKCM-Metastasis (Skin Cutaneous Melanoma-Metastasis), STAD (Stomach adenocarcinoma). PD-1**(A)** and CTLA-4 **(C)** are up-regulated in BRCA (Breast invasive carcinoma), LIHC (Liver hepatocellular carcinoma), LUAD (Lung adenocarcinoma), UCEC (Uterine Corpus Endometrial Carcinoma), while PD-L1**(B)** is significantly down-regulated in BRCA, LIHC, LUAD, and UCEC (*p < 0.05; **p < 0.01; ***p < 0.001).

**FIGURE 5:**
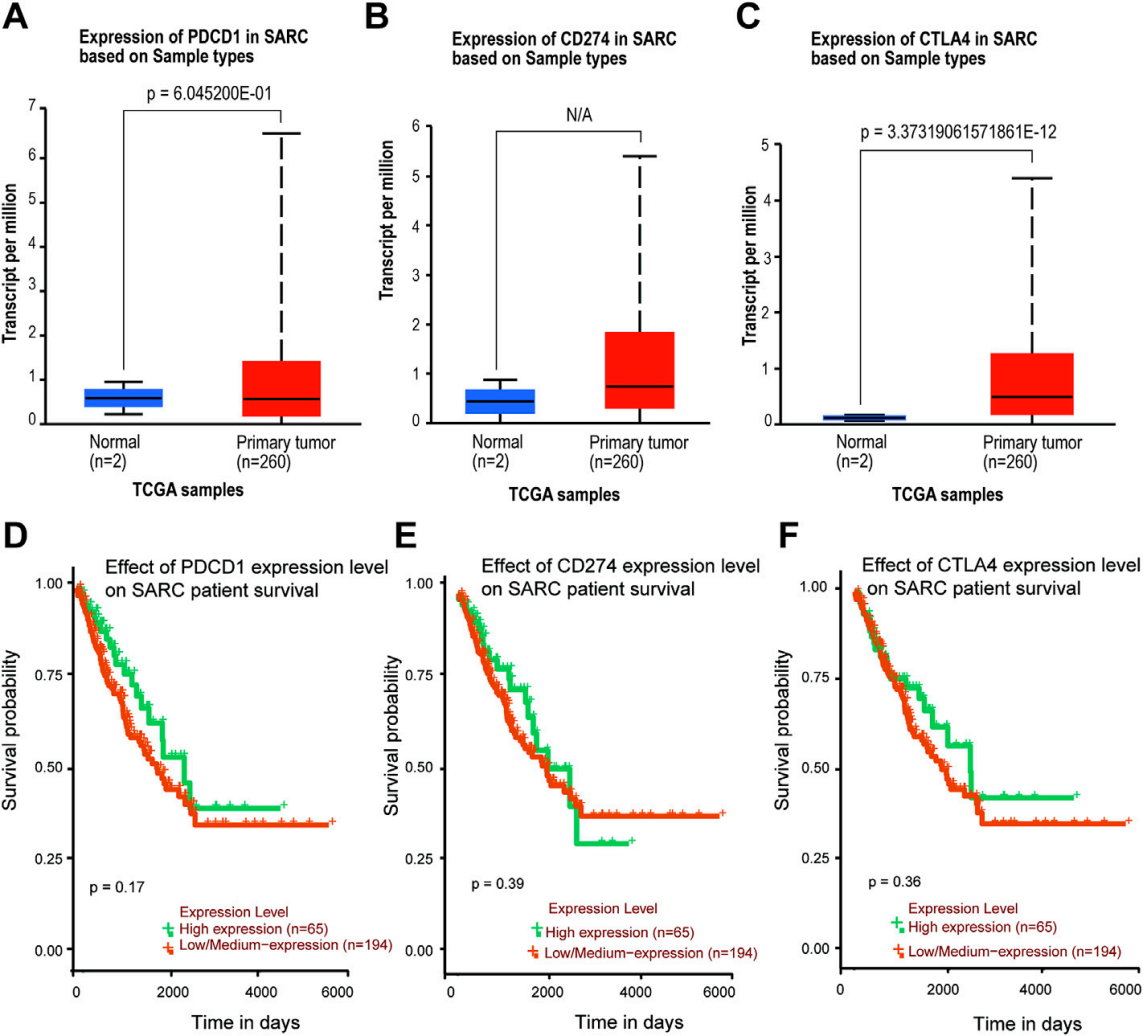
Expression level and survival probability of PD-1, PD-L1, CTLA-4 genes in SARC. **(A,B,C)** PD-1, PD-L1, CTLA-4 genes expression in SARC patients. Box plots showing distribution of tumor (Red) and paired non-tumor (Green) PD-1 **(A),** PD-L1 **(B)**, CTLA-4 **(C)** genes expression in SARC. **(D,E,F)** Kaplan–Meier survival curves comparing high and low/medium expression of PD-1 **(D)**, PD-L1 **(E)**, CTLA-4 **(F)** in SARC. Plots were generated using UALCAN tools (http://ualcan.path.uab.edu/) and curated manually.

The TIMER2 database is used to compare the expression levels of PD-1/PD-L1/CTLA-4 in pan-cancer and corresponding adjacent tissues. We detected that PD-1 and CTLA-4 are up-regulated in most tumor tissues, with statistical significance, including BRCA (Breast invasive carcinoma), CHOL (Cholangiocarcinoma), ESCA (Esophageal carcinoma), HNSC (Head and Neck squamous cell carcinoma), HNSC-HPV+ (Head and Neck squamous cell carcinoma- Human papillomavirus+), KIRC (Kidney renal clear cell carcinoma), KIRP (Kidney renal papillary cell carcinoma), LIHC (Liver hepatocellular carcinoma), LUAD (Lung adenocarcinoma), SKCM-Metastasis (Skin Cutaneous Melanoma-Metastasis), STAD (Stomach adenocarcinoma) and UCEC (Uterine Corpus Endometrial Carcinoma) (Figures 4A,C). All of them showed significantly up-regulation in CHOL, ESCA, HNSC, SKCM-Metastasis and STAD. However, PD-L1 is significantly down-regulated in BRCA, LIHC, LUAD, and UCEC (Figure 4B), which is different from PD-1 and CTLA-4. However, we did not find PD-1/PD-L1/CTLA-4 has any significant expression in SARC. Then the UALCAN database was used to compare the mRNA levels of PD-1/PD-L1/CTLA-4 between SARC and normal tissues (Figures 5A–C). We found that only the expression of CTLA-4 (Figure 5C) in SARC is higher than that in adjacent tissues, which is statistically significant. However, there is no obvious clinical significance through survival curve analysis (Figures 5D–F).

### 3.4 Immune infiltration analysis data

**FIGURE 6:**
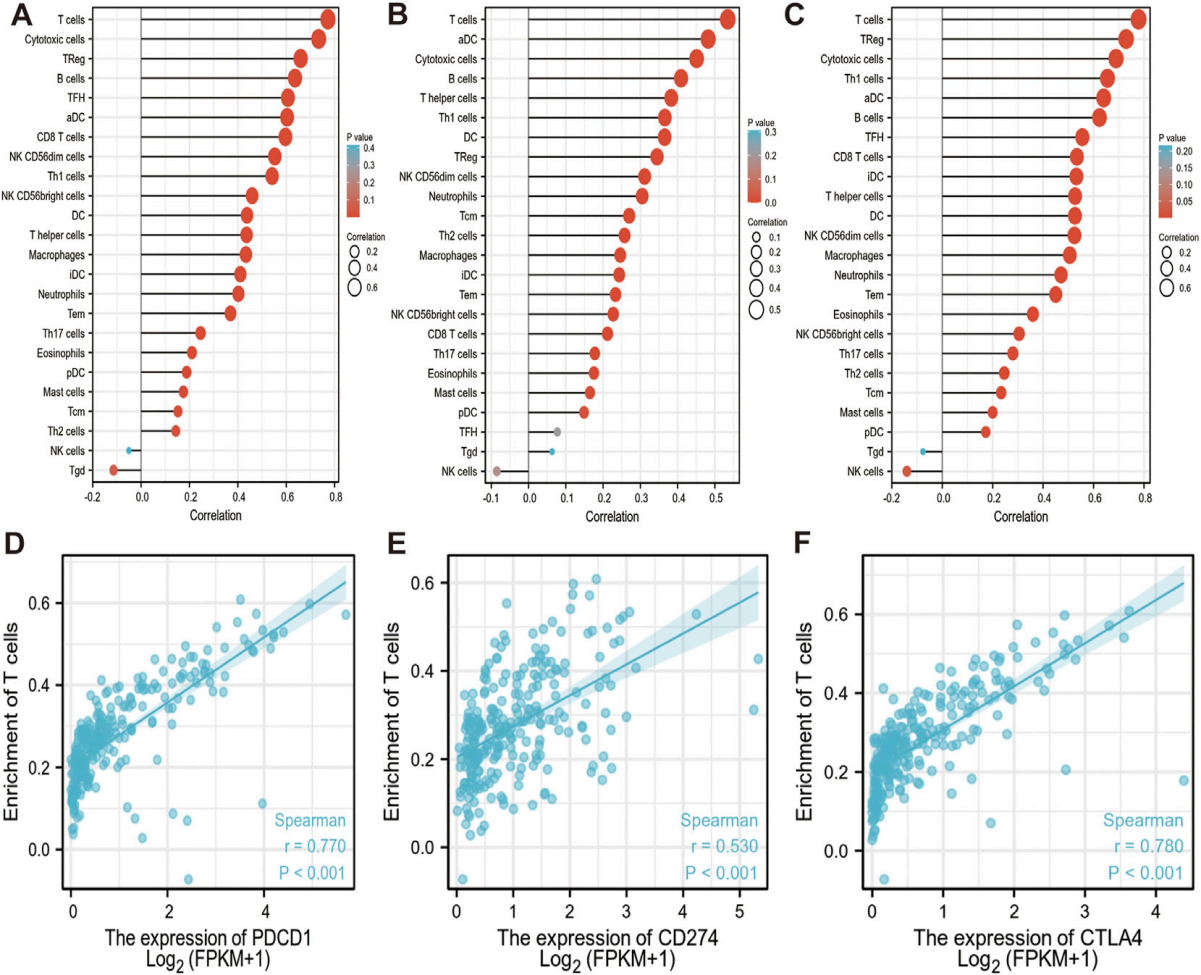
Correlation between PD-1/PD-L1/CTLA-4 expression and immune infiltration in SARC. **(A,B,C)** Correlation between the expression of PD-1 **(A)**, PD-L1 **(B)** and CTLA-4 **(C)** and the abundance of 24 tumor-infiltrating lymphocytes in SARC, computed using GSVA (https://www.bioconductor.org/packages/3.4/bioc/htmL/GSVA.html). Red circles represent positive correlations (Spearman_Cor>0). The size of circles is proportional to the spearman’s correlation co-efficient in immune cells. **(D,E,F)** Spearman correlation plots between log 2 PD-1 **(D)**, PD-L1 **(E)**, CTLA-4 **(F)** genes expression values and enrichment T cells are shown in SARC. Spearman correlation between the two measurements was positive significant (r > 0, *p* < 0.001). The blue lines represent the best-fit linear model, and the gray lines represent the 95% confidence interval, calculated using ggplot2 (https://cran.rproject.org/web/packages/ggplot2/index.html). aDC: active dendritic cells; B cells: B lymphocytes; CD8 T cells: Cytotoxic T lymphocytes; DC: dendritic cells; iDC: immature dendritic cells; NK CD56bright cells: natural killer CD56bright; NK CD56dim cells: natural killer CD56dim cells; pDC: Plasmacytoid dendritic cells; T cells: T lymphocyte; Tcm: T central memory; Tem: T effector memory; Tfh: T follicular helper; Th1 cells: T helper type 1 cells; Th17 cells: T helper 17 cells; Th2 cells: T Helper 2 Cells; Treg: Regulatory T cells.

As an important part of the tumor microenvironment, tumor-infiltrating immune cells are closely related to the occurrence, progression, or metastasis of cancer. The genes expression in immune cells data of sarcoma level 3 HTSeq-FPKM format RNAseq data were downloaded from the TCGA database for analysis. We found that PD-1, PD-L1, and CTLA-4 are up-regulated in these immune cells as aDC; B cells; CD8 T cells; cytotoxic cells; DC; eosinophils; iDC; macrophages; mast cells; neutrophils; NK CD56bright cells; NK CD56dim cells; pDC (Plasmacytoid DC); T cells; T helper cells; Tcm (T central memory); Tem (T effector memory); Tfh (T follicular helper); Th1 cells; Th17 cells; Th2 cells; and Treg (Figures 6A–C). Red circles represent positive correlations (Spearman_Cor>0) and the size of circles is proportional to the spearman’s correlation co-efficient in immune cells (Figures 6A–C). We found that PD-1/PD-L1/CTLA-4 was positively and significantly expressed on most immune cells, especially on T cells and active immune cells such as Treg, cytotoxic cells, aDC, T helper cells (Spearman_Cor>0, *p* < 0.05). Further analysis, we observed a strong positive correlation between the enrichment of T cells and the expression of PD-1, PD-L1 and CTLA-4 in SARC (*p* < 0.001, r > 0) (Figures 6D–F).

### 3.5 Co-expressed genes of PD-1/PD-L1/CTLA-4 analysis results

**FIGURE 7:**
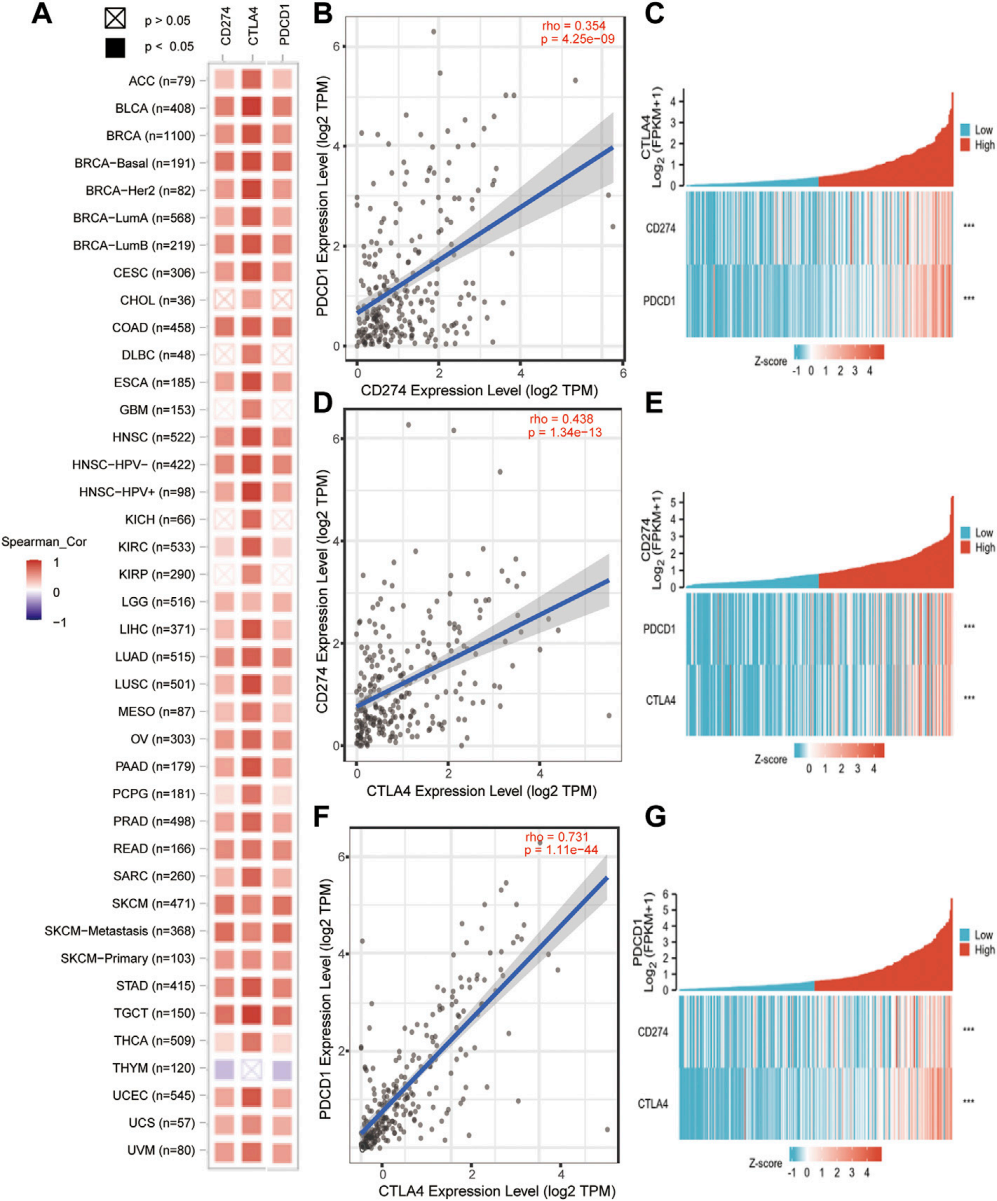
PD-1/PD-L1/CTLA-4 co-expressed in pan-cancer and SARC. **(A)** PD-1/PD-L1/CTLA-4 positively co-expressed in pan-cancer from TCGA database using TIMER2.0 tool (Spearman_ Cor>0, *p* < 0.05). **(B,D,F)** PD-1, PD-L1 and CTLA-4 positively correlated with each other in SRAC from TCGA database using TIMER2.0 tool (rho>0, *p* < 0.05). **(C,E,G)** The heatmap showed that PD-1, PD-L1, CTLA-4 were clearly co-expressed in SARC through co-expression clustering analysis using R package: mainly “ggplot2” (***p < 0.001).

In order to further understand the relationship of PD-1/PD-L1/CTLA-4, it is necessary to know the interaction of their co-expression in the tumor microenvironment. The TIMER2.0 method was used to analyze the correlation between the expression of PD-1, PD-L1, and CTLA-4 in pan-cancer, and it was detected that PD-1/PD-L1/CTLA-4 had a statistical correlation in pan-cancer (*p* < 0.05) (Figure 7A). Then use TIMER2.0 to find the correlation between each other in SARC, and found that they are all positively statistically correlated with each other (*p* < 0.05, rho>0) (Figures 7B,D,F). Furthermore, we used the data of SARC Level 3 HTSeq-FPKM format RNAseq to do co-expression clustering analysis, and the heatmap showed that PD-1, PD-L1, CTLA-4 were clearly co-expressed in SARC (***p < 0.001) (Figures 7C,E,G).

### 3.6 Protein-protein interaction and GO-KEGG pathway analysis data

**FIGURE 8:**
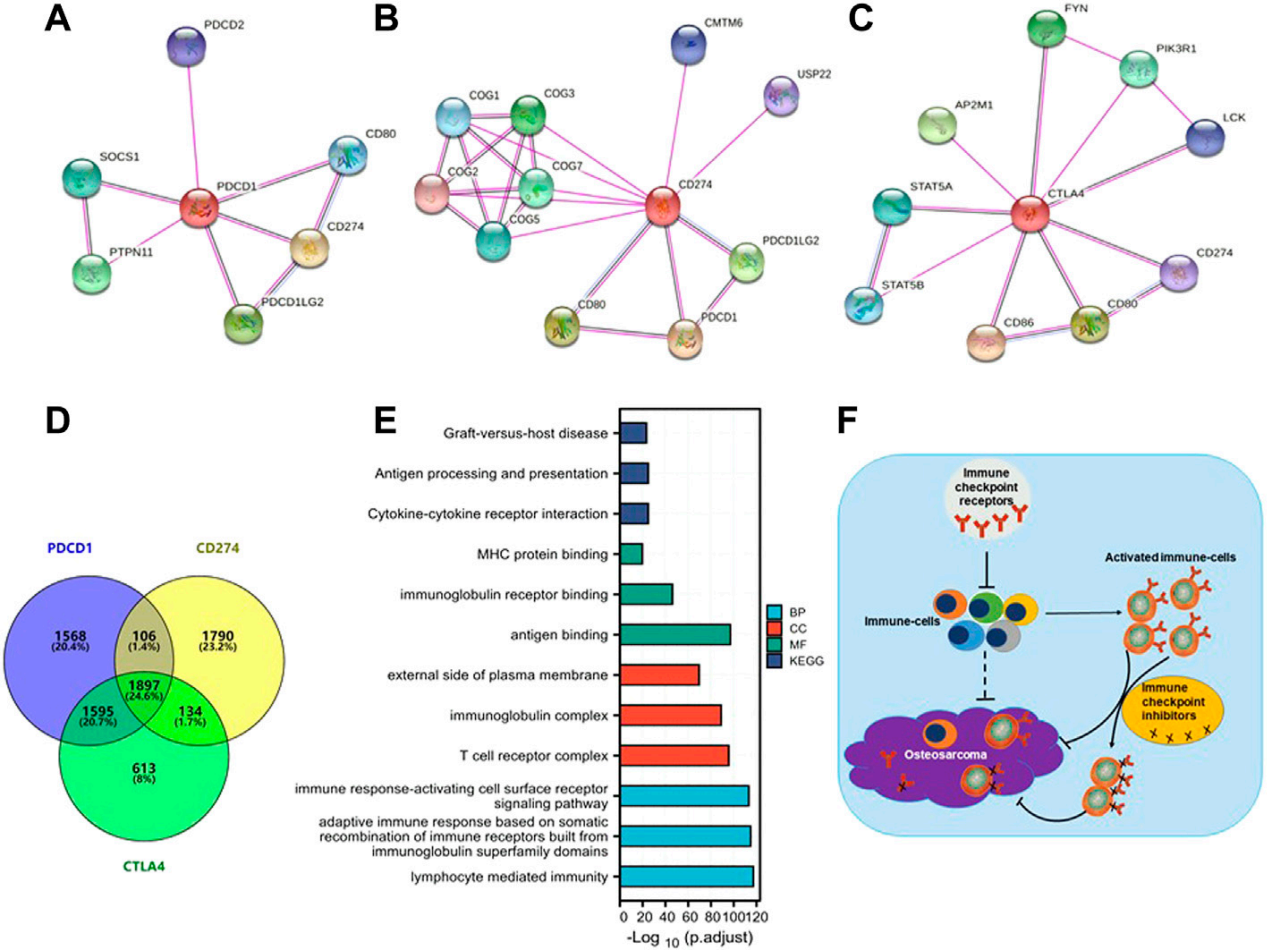
PD-1/PD-L1/CTLA-4 related gene enrichment analysis. **(A,B,C)** The top 10 hub genes in the PPI (Protein- Protein Interaction) network with high confidence using STRING tool. Circles represent different genes in the three modules, and interconnecting lines represent interactions between these genes. PD-1 **(A)**, PD-L1 **(B)** and CTLA-4 **(C)** are high-confidence hub genes for each other (circle logo). **(D)** Venn diagram showed that PD-1/PD-L1/CTLA-4 cross-co-expressed 1897 genes in SARC, accounting for the largest proportion of 24.6%. **(E)** Based on PD-1/PD-L1/CTLA-4 cross-co-expressed genes, GO-KEGG pathway enrichment analysis revealed co-expressed pathways. Gene Ontology (GO); Kyoto Encyclopedia of Genes and Genomes (KEGG); biological process (BP); molecular function (MF); cellular component (CC). **(F)** A Schematic inference model showing immune checkpoint receptors regulation of osteosarcoma. Immune checkpoint receptors inhibit the activation of immune cells; immune checkpoint receptors are mainly expressed on activated immune cells; and immune checkpoint inhibitors target activated immune cells to promote the killing of osteosarcoma.

To further study the molecular mechanism of the PD-1/PD-L1/CTLA-4 gene in tumorigenesis, attempts were made to screen out the target PD-1/PD-L1/CTLA-4 binding protein and expression related genes for a series of pathway enrichment analyses. Based on the STRING tool, PPI network analysis obtained PD-1/PD-L1/CTLA-4 binding top 10 proteins, which is supported by experimental evidence. Figures 8A–C demonstrates the interaction network of these proteins. It was found that these genes are high-confidence hub genes for each other.

The SARC level 3 HTSeq-FPKM format RNAseq data downloaded from TCGA to do pathway enrichment analysis was used, co-expressed genes with *p* < 0.001 were selected. Thus, 3932, 4256, and 5175 co-expressed genes with PD-1, PD-L1, and CTLA-4, respectively, were selected. The Venn diagram demonstrates that 1897 genes are intersection co-expressed, accounting for 24.6% of the total genes, with the highest proportion (Figure 8D).

Using the intersection of co-expressed genes from the SARC level 3 HTSeq-FPKM format RNAseq data from TCGA, the GO-KEGG pathway analysis was conducted (Figure 8E; Table 2). Enrichment was related to the significance of the specific function. From GO analysis, these genes were strikingly enriched in three categories: (1) BP: lymphocyte-mediated immunity (GO: 0002449), adaptive immune response based on somatic recombination of immune receptors built from immunoglobulin superfamily domains (GO: 0002460), immune response-activating cell surface receptor signaling pathway (GO: 0002429); (2) CC: T cell receptor complex (GO: 0042101), immunoglobulin complex (GO: 0019814), external side of the plasma membrane (GO: 0009897); (3) MF: antigen binding (GO:0003823), immunoglobulin receptor binding (GO: 0034987), MHC protein binding (GO: 0042287). We further performed the KEGG pathway analysis of target genes. The enriched pathways were cytokine-cytokine receptor interaction (hsa04060), antigen processing and presentation (hsa04612), and graft-versus-host disease (hsa05332).

**TABLE 2:** GO-KEGG analysis of PD-1/PD-L1/CTLA-4 intersection co-expression genes.

Ontology	Id	Description	GeneRatio	BgRatio	*p*-value	p.adjust	q-value
BP	GO:	Lymphocyte mediated immunity	186/1299	352/18670	8.42e-122	4.23e-118	3.36e-118
	0002449						
BP	GO:	Adaptive immune response based on somatic recombination of immune receptors built from immunoglobulin superfamily domains	186/1299	361/18670	3.62e-119	9.10e-116	7.22e-116
	0002460						
BP	GO:	Immune response-activating cell surface receptor signaling pathway	209/1299	473/18670	2.90e-117	4.85e-114	3.85e-114
	0002429						
CC	GO:	T cell receptor complex	104/1348	127/19717	3.77e-99	1.90e-96	1.61e-96
	0042101						
CC	GO:	Immunoglobulin complex	111/1348	159/19717	3.64e-92	9.19e-90	7.79e-90
	0019814						
CC	GO:	External side of plasma membrane	149/1348	393/19717	1.37e-72	2.30e-70	1.95e-70
	0009897						
MF	GO:	Antigen binding	116/1184	160/17697	8.24e-101	6.94e-98	6.14e-98
	0003823						
MF	GO:	Immunoglobulin receptor binding	56/1184	76/17697	1.46e-49	6.13e-47	5.42e-47
	0034987						
MF	GO:	MHC protein binding	27/1184	40/17697	7.56e-23	1.82e-20	1.61e-20
	0042287						
KEGG	hsa04060	Cytokine-cytokine receptor interaction	85/630	295/8076	4.39e-28	1.14e-25	8.92e-26
KEGG	hsa04612	Antigen processing and presentation	43/630	78/8076	8.03e-28	1.14e-25	8.92e-26
KEGG	hsa05332	Graft-versus-host disease	31/630	42/8076	4.26e-26	4.05e-24	3.15e-24

In conclusion, our study elucidates that differential expression of PD-1/PD-L1/CTLA-4 on sarcoma cells is not an independent prognostic factor in sarcoma nor mutation frequency. PD-1/PD-L1/CTLA-4 was not consistently expressed in the same direction in all tumors; however, the direction of expression change was the same in most immune cells. The expression of PD-1/PD-L1/CTLA-4 on tumors may not be an effective target of immune checkpoint inhibitors, but is related to immune infiltration in the immune microenvironment (Figure 8F).

## 4 Discussion

Immune checkpoint inhibitors are tumor immunotherapy drugs that activate the immune system to fight tumor cells by blocking checkpoint receptors, primarily including CTLA-4 and PD-1 or its main ligand PD-L1. Immune checkpoint inhibitor PD-1/PD-L1/CTLA-4 has conducted extensive research on some tumors such as melanoma. Clinical trials found that the therapeutic effect of an immune checkpoint inhibitor in malignant melanoma was significantly better than that of traditional therapy and was approved by Food and Drug Administration. Subsequent studies detected that it was also effective in non-small cell lung cancer, ovarian cancer, and renal-cell cancer (Bagchi et al., 2021). Currently, there are primarily several kinds of immune checkpoint inhibitors: Anti-PD-1: Nivolumab and pembrolizumab, Anti-PD-L1: Atezolizumab, Durvalumab, Avelumab; and Anti-CTLA-4: Ipilimumab. The subsequent study swept almost all the common malignant tumors; however, the results of clinical trials did not fulfill the expectations (Table 3). Compared with the traditional treatment, particularly in osteosarcoma, the effect is even less satisfactory. Furthermore, Dr. Suzanne George stressed that the role of pembrolizumab (Anti-PD-1) in the treatment of osteosarcoma is quite limited. Although several immune checkpoint inhibitors have achieved good results in other tumors, a recent systematic retrospective analysis found that in osteosarcoma, compared with chemotherapy, it is far from achieving the effective rate of combined chemotherapy (Gazouli et al., 2021). The difference in treatment is so huge that we urgently need to conduct comprehensive analysis and research from basic experiments, specific mechanisms to clinical practice.

**TABLE 3:** Clinical trials of CTLA-4 and PD-1/PD-L1 blocking antibodies in human malignancies.

Target	Phase	Antibody	Tumor type	No. patients	Effective rate	Nct number	Ref
CTLA-4	III	Lpilimumab/cancer vaccine (gp100)	Unresectable stage III or IV melanoma	676	Prolonged overall survival rate	NCT00094653	Hodi et al. (2010)
PD-1	I	BMS-936558 (also known as MDX-1106 and ONO-4538)	Non–small-cell lung cancer	76	18%	NCT00730639	Topalian et al. (2012)
			Melanoma	94	28%		
			Renal-cell cancer	33	27%		
	Pediatric	Nivolumab	Osteosarcoma	13	No significant single-agent activity	NCT02304458	Davis et al. (2020)
	I /II						
	II	Pembrolizumab	Osteosarcoma, Ewing sarcoma, Chondrosarcoma	40	2.5% (1/40)	NCT02301039	Tawbi et al. (2017)
	II	Pembrolizumab plus cyclophosphamide	Advanced osteosarcoma	15	13.3% (2/15)	NCT02406781	Le Cesne et al. (2019)
	II	Pembrolizumab	Advanced osteosarcoma	12	0%	NCT03013127	Boye et al. (2021)
PD-L1	I	BMS-936559	Non–small-cell lung cancer	75	10.2% (5/49)	NCT00729664	Brahmer et al. (2012)
			Melanoma	55	17.3% (9/52)		
			Colorectal cancer	18	/		
			Renal-cell cancer	17	11.7% (2/17)		
			Ovarian cancer	17	5.8% (1/17)		
			Pancreatic cancer	14	/		
			Gastric cancer	7	/		
			Breast cancer	4	/		

PD-1, PD-L1, CTLA-4 are the main immune checkpoint receptor genes, which are mainly expressed in activated T cells and various tumor cells, while at the same time inhibit the activation of T cells and participate in the regulation of T cell function. The expression levels of immune checkpoint receptors determine the activation or exhaustion state of immune cells. PD-1, an immunosuppressive receptor, is expressed in activated T cells and various tumor cells and participates in the regulation of T cell functions, including the function of effector CD8^+^ T cells (Sun et al., 2018). PD-L1 encoding an immunosuppressive receptor ligand is expressed by hematopoietic and non-hematopoietic cells, such as T cells, B cells, and various tumor cells. PD-L1 is a type I transmembrane protein with immunoglobulin type V and C domains. The interaction of PD-L1 with its receptor inhibits T cell activation and cytokine production. In the tumor microenvironment, this interaction provides an immune escape for tumor cells through the inactivation of cytotoxic T cells. CTLA-4 is a member of the immunoglobulin superfamily, which encodes a protein that transmits inhibitory signals during T cell activation. CTLA-4 gene is expressed in a variety of cells, including B cells, monocytes, granulocytes, CD34^+^ stem cells, and various tumor cells. However, CTLA-4 is primarily expressed on activated T cells. Resting T cells rarely express CTLA-4.

In this study, we found that these three genes have Ig-like V-type protein domain, whereas PD-1 and CTLA-4 are strikingly similar with only one Ig-like V-type protein domain (Figure 1), located on the same chromosome 2q (Table 1). Protein structure largely determine its function (Figure 4), and these observations found that PD-1/PD-L1/CTLA-4 was up-regulated in CHOL, ESCA, HNSC, and SKCM-Metastasis, STAD tumors. PD-1 and CTLA-4 were consistently up-regulated in most tumors, including BRCA, CHOL, ESCA, HNSC, HNSC-HPV+, KIPC, KIRP, LIHC, LUAD, SKCM-Metastasis, STAD and UCEC. The structural differences between PD-L1 and PD-1, CTLA-4, we observed that the expression of the same tumor was significantly different. PD-L1 expression was down-regulated in BRCA, LIHC, LUAD, and UCEC, while PD-1, CTLA-4 expression was up-regulated in these tumors. The significantly different expressions on the same tumor indicate that their distribution on the tumor is not the same. The difference is whether they are differentially expressed on activated T cells on the tumor or on the tumor itself. Through further analysis, we found that their expression levels on the immune cells of SARC were surprisingly consistent (Figure 6), and there is reason to suspect that their differential expression depends on the tumor itself.

We found that although the expression levels of PD-1 and PD-L1 in sarcoma and normal tissues were not significantly different, only the expression of CTLA-4 was different, but there was no significant difference in survival probability (Figure 5). Further analysis of their mutation levels on sarcomas shows that their mutation levels are not high, only PD-1 is slightly higher by 8%, and the types of mutations are mainly missense, and further analysis of mutations and the disease-free survival rate of patients have no statistics significance (Figure 2, Figure 3). It shows that mutation is not the main factor in determining its outcome.

Although there was no clear relationship between PD-1/PD-L1/CTLA-4 gene expression and prognosis in SARC patients, their immune cell infiltration in SARC was consistent (Figure 6). Through the co-expression gene analysis of these three genes in TCGA database, it was found that they have a considerable number of co-expressed genes in SARC, accounting for 25.6%, which is the highest proportion among all expressions (Figure 8). PPI network protein interaction analysis and further enrichment analysis of cross-co-expressed genes by GO-KEGG pathway showed that co-expressed genes were mainly enriched in immune response pathways (Figure 8), and indicates that these three genes are closely related to the immune microenvironment of sarcoma. From the analysis of the results of numerous clinical trials (Table 3), it is concluded that the therapeutic effects of immune checkpoint inhibitors on different tumors are significantly different. Due to the dramatic effect in malignant melanoma, we do not doubt the lethality of immune checkpoint inhibitors against malignancies, but it may be more important to consider that the therapeutic effect of immune checkpoint inhibitors depends on targeting activated immune cells on the tumor. Therefore, it is speculated from our findings that how to transport activated immune cells into osteosarcoma tissue is the key to treatment. Although immune checkpoint inhibitors have made great achievements in malignant melanoma, the final results may be very different due to the different immune microenvironment and pathological characteristics of each tumor. This study still has some limitations: it is only based on the conclusions drawn from the large number analysis, and further experiments are needed to verify the specific synergistic effect of these three genes in the osteosarcoma immune microenvironment. Although the results of clinical trials in osteosarcoma have been very disappointing, immunotherapy is undoubtedly a very promising cancer treatment.

## Data Availability

Publicly available datasets were analyzed in this study. This data can be found here: https://www.ncbi.nlm.nih.gov/gene/, PDCD1, CD274, CTLA4.
